# An intestinal stem cell niche in *Apc* mutated neoplasia targetable by CtBP inhibition

**DOI:** 10.18632/oncotarget.25784

**Published:** 2018-08-21

**Authors:** Ayesha T. Chawla, Agnes D. Cororaton, Michael O. Idowu, Priyadarshan K. Damle, Barbara Szomju, Keith C. Ellis, Bhaumik B. Patel, Steven R. Grossman

**Affiliations:** ^1^ VCU Wright Center for Clinical and Translational Research, Virginia Commonwealth University, Richmond, VA 23298, USA; ^2^ Department of Internal Medicine, Virginia Commonwealth University, Richmond, VA 23298, USA; ^3^ Department of Pathology, Virginia Commonwealth University, Richmond, VA 23298, USA; ^4^ VCU Massey Cancer Center, Virginia Commonwealth University, Richmond, VA 23298, USA; ^5^ Department of Medicine, Hunter Holmes McGuire VA Medical Center, Richmond, VA 23249, USA; ^6^ Department of Medicinal Chemistry, Virginia Commonwealth University, Richmond, VA 23298, USA

**Keywords:** c terminal binding protein, adenomatous polyposis, tumor initiating cells

## Abstract

C-terminal binding protein 2 (CtBP2) drives intestinal polyposis in the *Apc* ^*min*^ mouse model of human Familial Adenomatous Polyposis. As CtBP2 is targetable by an inhibitor of its dehydrogenase domain, understanding CtBP2’s role in adenoma formation is necessary to optimize CtBP-targeted therapies in *Apc* mutated human neoplasia. Tumor initiating cell (TIC) populations were substantially decreased in *Apc* ^*min*^ *Ctbp2*^*+/-*^ intestinal epithelia. Moreover, normally nuclear Ctbp2 was mislocalized to the cytoplasm of intestinal crypt stem cells in *Ctbp2*^*+/-*^ mice, both *Apc* ^*min*^ and wildtype, correlating with low/absent CD133 expression in those cells, and possibly explaining the lower burden of polyps in *Apc* ^*min*^ Ctbp2^+/-^ mice. The CtBP inhibitor 4-chloro-hydroxyimino phenylpyruvate (4-Cl-HIPP) also robustly downregulated TIC populations and significantly decreased intestinal polyposis in *Apc* ^*min*^ mice. We have therefore demonstrated a critical link between polyposis, intestinal TIC’s and *Ctbp2* gene dosage or activity, supporting continued efforts targeting CtBP in the treatment or prevention of *Apc* mutated neoplasia.

## INTRODUCTION

Sporadic colon cancer is frequently characterized by mutation of the *APC* tumor suppressor, while autosomal dominant inheritance of a mutant *APC* allele, as in Familial Adenomatous Polyposis (FAP), results in early onset massive colonic polyposis that uniformly progresses to colorectal cancer unless prophylactic total colectomy is performed [1]. Recently, we have shown that the emerging oncogene and drug target, C-terminal binding protein 2 (CtBP2), is a key driver of neoplasia in the *Apc* ^*min/+*^ (*Apc* ^*min*^) mouse model of human FAP [2].

C-terminal binding proteins 1 and 2 (CtBP) are paralogous transcriptional co-regulators frequently overexpressed and associated with worse outcome and aggressive tumor features in colon, [3] breast [4, 5], gastric [6], ovarian [7], and prostate [8] cancers. Indeed, the majority of colon and breast tumors demonstrate overexpression of CtBP1 and/or CtBP2 proteins (64% [3] and 92% [4], respectively). CtBP upregulation leads to inappropriate cell survival as well as enhanced migratory and invasive properties due to the ability of CtBP to repress transcription of *Bik* [9], *Brca1* [4], *PTEN* [10], the epithelial adhesion protein *E-cadherin* [11], and many other tumor suppressive genes [11, 12], as well as co-activate the migration-associated gene *Tiam1* [13] and the drug efflux pump *MDR1* [14]. *In vitro* overexpression of CtBP is oncogenic in a manner similar to mutant H-Ras [2], transforming primary mouse embryo fibroblasts to anchorage-independent growth, which is a strong predictor of tumor growth in mouse xenograft models [15]. Importantly, CtBP transcriptional co-regulation is activated by an increase in NADH concentration, as is often the case in hypoxic and/or glycolytically active tumors [16], due to NADH-dependent oligomerization of CtBP’s conserved dehydrogenase domain [17].

The functional dehydrogenase domain encoded by CtBP1/2 is targetable by small molecule analogues of its native substrate α-keto-γ-(methylthio) butyric acid (MTOB). Of these analogues, 2-hydroxyimino-3-phenyl-propionic acid (HIPP) and its more potent 4-chloro-derivative (4-Cl-HIPP), antagonize CtBP’s proposed oncogenic functions [18]. Pharmacological inhibition of CtBP using HIPP profoundly reduced intestinal polyposis in *Apc* ^*min*^ mice, similarly to haploinsufficiency of *Ctbp2* [2], and with no observable toxicity.

CtBP2’s role in driving a tumor initiating cell (TIC) niche in solid tumors is emerging [19]. TICs contribute to intra-tumoral heterogeneity, metastasis and chemoresistance in a variety of solid cancers, including colon, pancreatic and ovarian, among other cancers [20–24], and an ideal therapy would target this population to overcome metastatic or local relapse from treatment-resistant TICs. Both normal stem cell and TIC populations from intestinal epithelia exhibit the same cell surface markers, though underlying molecular events, such as *APC* allelic loss, transform normal intestinal stem cells into TIC’s [21, 25]. A number of TIC-related cell surface markers have been identified in the intestine, including CD44, CD24, CD133, and CXCR4 (23, 28-32). CD44+/CD24+ populations obtained from colorectal tumors can initiate growth of colonospheres *in vitro* and colorectal tumors *in vivo*, and similar cells exhibit TIC activity across a variety of solid tumors [26]. The progeny of CD133+ cells are capable of giving rise to all major intestinal cell types, as well as neoplasms [21, 27], and CD133+ marks long-lived multipotent intestinal stem cells [28]. CXCR4, a chemokine receptor, is expressed both in normal tissue stem cells of the breast, lung, and prostate gland, as well as tumors formed in those organs [29–32] Consistent with a role of colon TIC’s, colon cancer micrometastases require CXCR4 to initiate proliferation, and CXCR4/CD133 dual positive cells demonstrate enhanced tumorigenic capabilities over unsorted cells [33, 34]. CXCR4 is also found to be overexpressed in the majority of colon cancer cases [35, 36].

In this work we have interrogated the effect of Ctbp2 haploinsufficiency on the intestinal stem cell niche of both wild type and *Apc* ^*min*^ mice, to better understand Ctbp2’s biologic role in the expanded stem cell population in *Apc* ^*min*^ mice that serve as precursors to polyps, which are enriched for cells with stem cell-like markers that are characterized as tumor initiating cells (TIC’s). We show that *Ctbp2* loss or inhibition with 4-Cl-HIPP both reduce normal stem cell and TIC populations in *Apc* ^*min*^ intestine, with a surprising finding of Ctbp2 protein misolocalization to the cytoplasm of stem cells and TIC’s when haploinsufficient. This mislocalization could explain the profound effect of Ctbp2 haploinsufficiency on polyp number and survival in *Apc* ^*min*^ mice and supports further therapeutic development of CtBP as a target in *APC* mutated neoplasia.

## RESULTS

### Ctbp2 gene dosage determines TIC abundance in *Apc* ^*min*^ intestine

**Figure 1:**
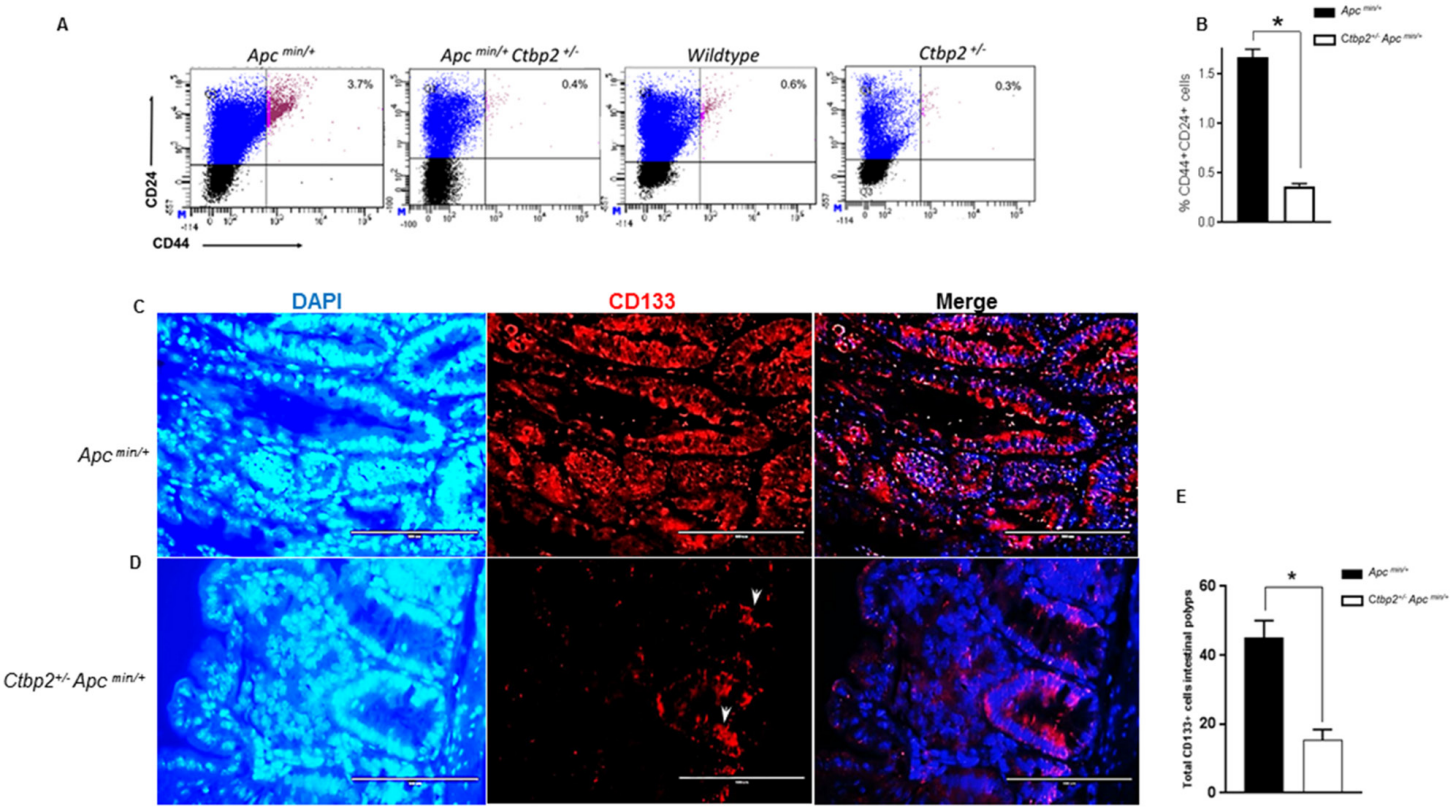
*Ctbp2* haploinsufficiency decreases TIC populations in *Apc*^*min/+*^ intestinal epithelia **(A)** Scatter plots of representative flow cytometric analyses of intestinal epithelial cells for CD44 and CD24 with top right quadrants representing percentage of CD44+CD24+ cells in age-matched mice of indicated genotypes. **(B)** Quantification of CD44+CD24+ cells from intestinal epithelia of indicated genotypes; n=3 biologic replicates. ^*^ p<0.05 for all analyses, error bars represent standard deviation from the mean. **(C, D)** IF staining for CD133+ cells on paraffin sections of intestinal polyps from age-matched (4 months) mice of indicated genotypes using anti-CD133 antibodies followed by Alexa flour 594 secondary antibody and DAPI stain (blue) to define nuclei; representative CD133 positive cells in Ctbp2 heterozygous polyps indicated by arrows. **(E)** Total CD133 positive cells in mouse small intestine obtained from indicated genotypes (n= 3 polyps/ mouse, ^*^p<0.05, error bars represent standard deviation from the mean).

To understand the role of Ctbp2 specifically in TIC activity induced by *Apc* mutated neoplasia, we first compared CD44+/CD24+ as well as CD133+/CXCR4+ populations (which include both normal stem cells and TIC’s) in small intestinal epithelia obtained from *Apc* ^*min/+*^, *Ctbp2* ^*+/-*^ *Apc* ^*min/+*^, *wildtype* and *Ctbp2* ^*+/-*^ mice (Figure 1A, Supplementary Figure 1A). Dual positive CD44+/CD24+ and CD133+/CXCR4+ cells were at least 2-fold less abundant in *Ctbp2*^*+/-*^ *Apc* ^*min/+*^ compared with age matched *Apc* ^*min/+*^ epithelia (Figure 1B, Supplementary Figure 1B). CD24+/CD44+ and CD133+/ CXCR4+ normal stem cell populations were also decreased 2-fold in non-neoplastic *Ctbp2*^*+/-*^ compared with *wildtype* intestinal epithelia (Figure 1A), indicating control of Ctbp2 over both normal and neoplastic (TIC) stem cell populations.

Histologic examination of CD133 expression by immunofluorescence in age matched *Apc* ^*min/+*^ vs. *Ctbp2* ^*+/-*^ *Apc* ^*min/+*^ mouse small intestinal polyps (Figure 1C-1D) suggested that CD133 expression was abundant, although not consistent, throughout the adenomatous polyps of *Apc* ^*min/+*^ mice, as has been reported [27] (Figure 1C). Interestingly, the few adenomatous polyps from *Ctbp2* ^*+/-*^ *Apc* ^*min/+*^ mice exhibited significantly diminished CD133+ expression (Figure 1D-1E), along with markedly less proliferative potential, as determined by Ki-67 staining, towards the edge of the polyp (Supplementary Figure 1C), as compared with *Apc* ^*min/+*^ adenomas.

**Figure 2:**
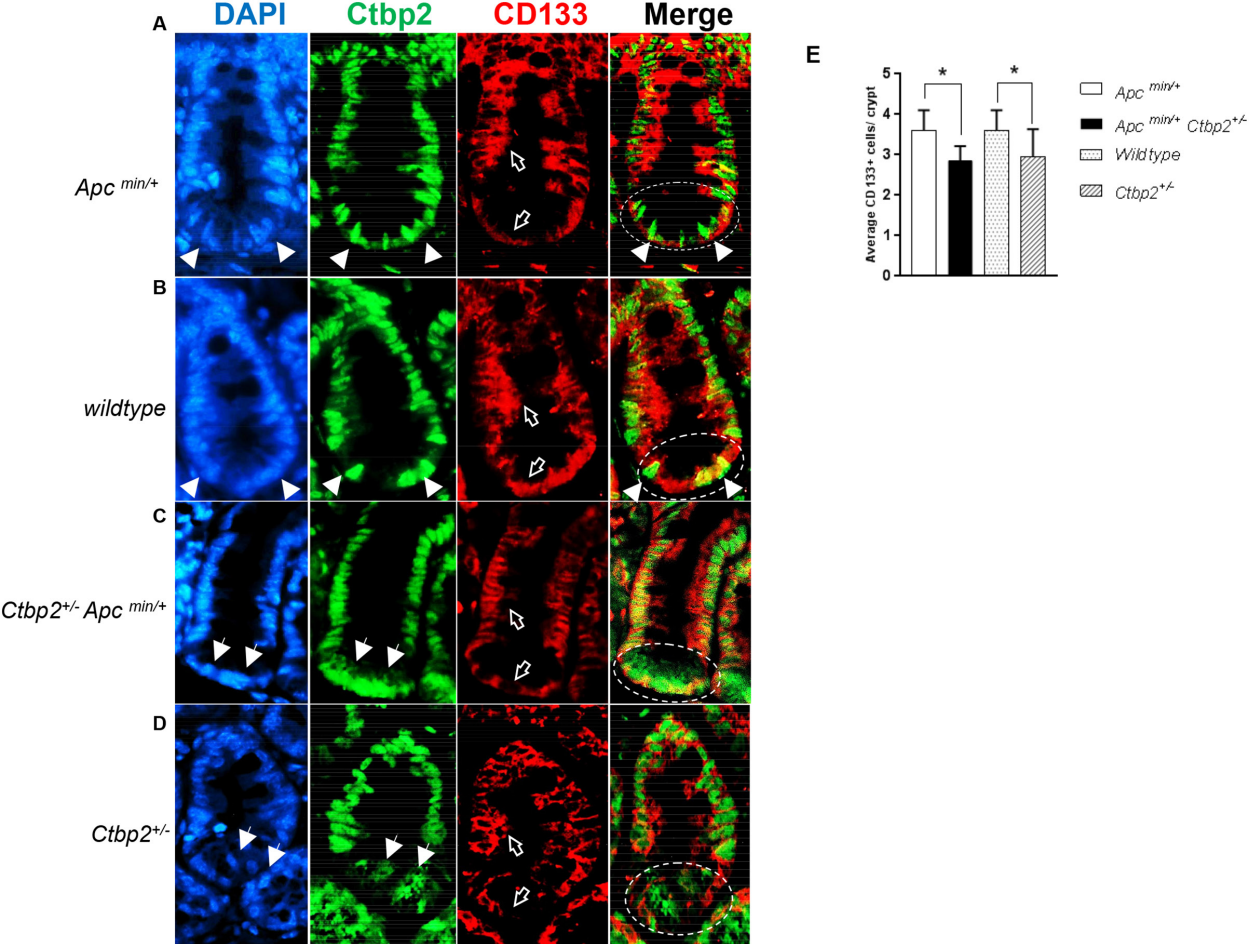
CD133 expression is ablated in *Ctbp2* haploinsufficient intestinal crypts **(A-D)** IF staining of normal small intestinal crypts for CD133 (red, empty arrows) and Ctbp2 (green, filled arrows) using anti-CD133 and anti-Ctbp2 antibodies followed by Alexa flour 594 and Cruz flour 488 secondary antibodies on paraffin sections from age-matched (4 months) mice of indicated genotypes. DAPI stain (blue) defines nuclei. The stem cell zone is represented in circles in the Merge view. Magnification = 400X. **(E)** Average CD133 positive cells per small intestinal crypt from each genotype in (A-D) determined by counting CD133+ cells from 2 mice of each genotype and 10 crypts per mouse intestine, ^*^p value <0.005, error bars represent standard deviation from the mean.

Expression of CD133 in normal crypts of aforementioned genotypes revealed that CD133 was equivalently expressed throughout the lower half of the intestinal crypts of *Apc* wildtype and *Apc* ^*min*^ small intestines (See empty arrows, CD133 panel, Figure 2A-2D, 2E), especially in transit amplifying cells and early progenitors, as previously reported [27].

Consistent with Ctbp2’s nuclear role in coregulation of Wnt target genes [37], Ctbp2 expression in *Apc* ^*min*^ (See arrows, DAPI and Ctbp2 panels, Figure 2A) and *Apc* wildtype (See arrows, DAPI and Ctbp2 panels, Figure 2B) normal crypt intestinal stem cells, or the “stem cell zone” (See circled area indicating stem cell zone in Merge panel, Figure 2A), was robust and predominantly nuclear (See arrows indicating nuclear Ctbp2 in Merge panel, Figure 2A). Staining for Ctbp2 in normal intestinal crypts from Ctbp2 haploinsufficient genotypes (*Apc* ^*min*^ or *Apc WT*) demonstrated markedly less nuclear Ctbp2 expression and mostly cytoplasmic expression in the stem cell zone (See arrows, DAPI and Ctbp2 panels Figure 2C and 2D). Moreover, the stem cell zone of *Ctbp2* haploinsufficient crypts where cytoplasmic Ctbp2 was observed, also showed partial loss of CD133 expression (See empty arrows in CD133 panel and circles in Merge panel, Figure 2C and 2D), with the average number of CD133+ cells in *Ctbp2*^*+/-*^ crypts significantly lower than in *Ctbp2*^*+/+*^ crypts (Figure 2E). This indicates a tight correlation between stemness (i.e. CD133 expression) and Ctbp2 nuclear localization, suggesting that the decrease in polyps in *Ctbp2*^*+/-*^ *Apc*^*min/+*^ mice [2] and the lower abundance of stem cells in Ctbp2 haploinsufficient polyps (Figure 1D-1E), may, in part, be due to cytoplasmic mislocalization of Ctbp2 that correlates with loss of crypt stemness (Figure 2C-2D).

**Figure 3:**
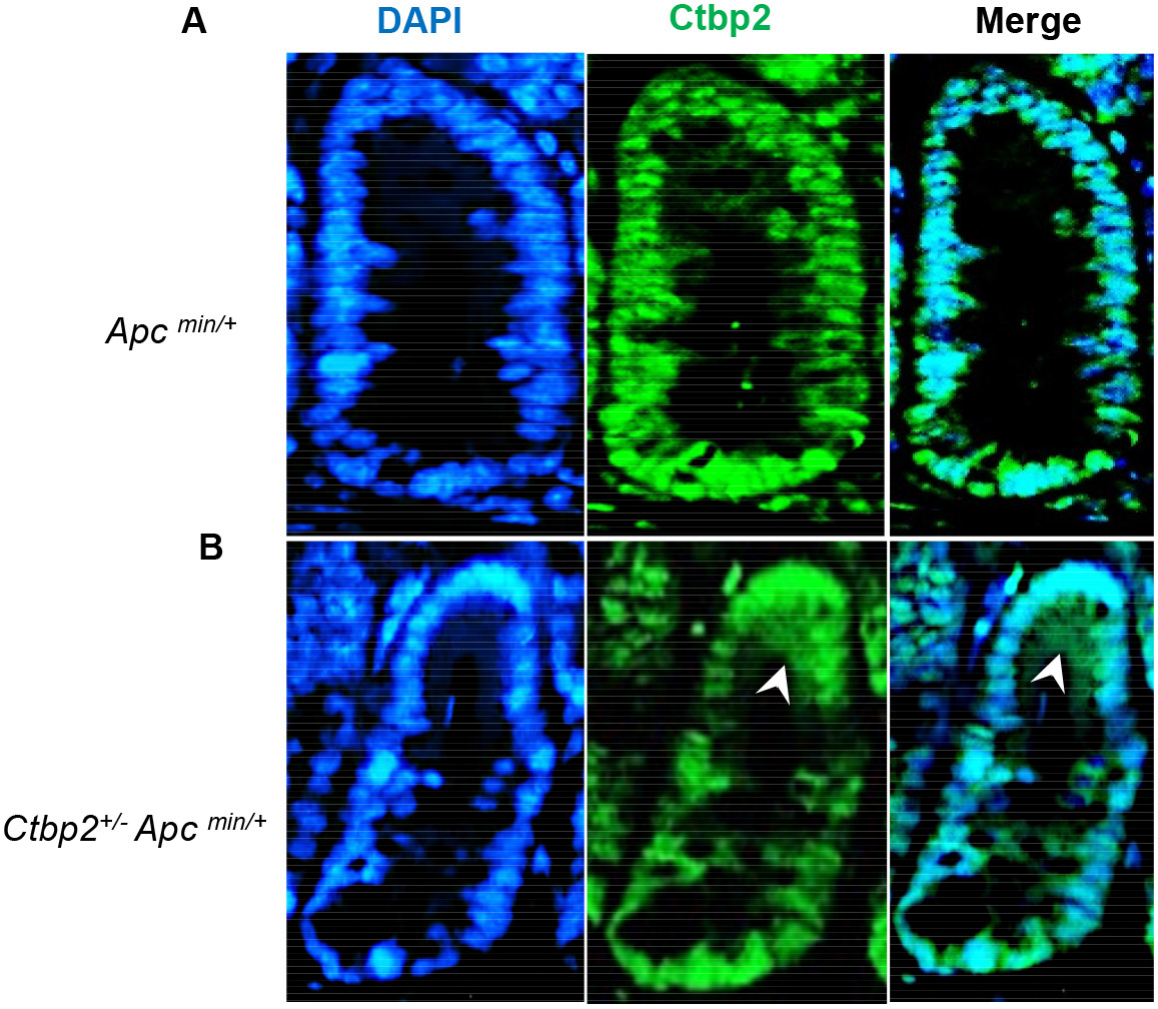
Ctbp2 localization in Ctbp2 wildtype vs. Ctbp2 haploinsufficient *Apc* ^*min*^ small intestinal polyps Ctbp2 expression (green) was determined by IF with anti-Ctbp2 antibody and Cruz flour 488-conjugated secondary antibody in small intestinal polyps from age-matched (4 months) **(A)** *Apc* ^*min/+*^ and **(B)** *Ctbp2*^*+/-*^ *Apc* ^*min/+*^ mice. Arrows indicate cells with cytoplasmic Ctbp2 localization in (B). DAPI (blue) stain indicates nuclei. Magnification = 400X.

The expression of CD133 was otherwise intact in the stem cell zone of intestinal crypts from *Ctbp2* WT mice (both *Apc* ^*min*^ and *Apc* wildtype), suggesting that both alleles of *Ctbp2* must be present to sustain expression of CD133 in the stem cell zone. By inference, there is a need for a full complement of Ctbp2 to maintain a phenotypically normal crypt stem cell compartment. Also consistent with this finding in normal crypts, Ctbp2 was predominantly cytoplasmic in adenomatous cells from *Ctbp2*^*+/-*^ *Apc*^*min/+*^ small intestinal polyps as compared with its usual nuclear expression in *Ctbp2*^*+/+*^ *Apc*^*min/+*^ polyps (Figure 3A-3B), suggesting the mechanism that led to Ctbp2 cytoplasmic localization in normal crypt stem cells, carried forward when stem cells transformed to form TIC’s and adenomatous polyps.

### Pharmacological inhibition of Ctbp limits polyposis and intestinal stem cell/TIC abundance in *Apc* ^*min*^ mice

**Figure 4:**
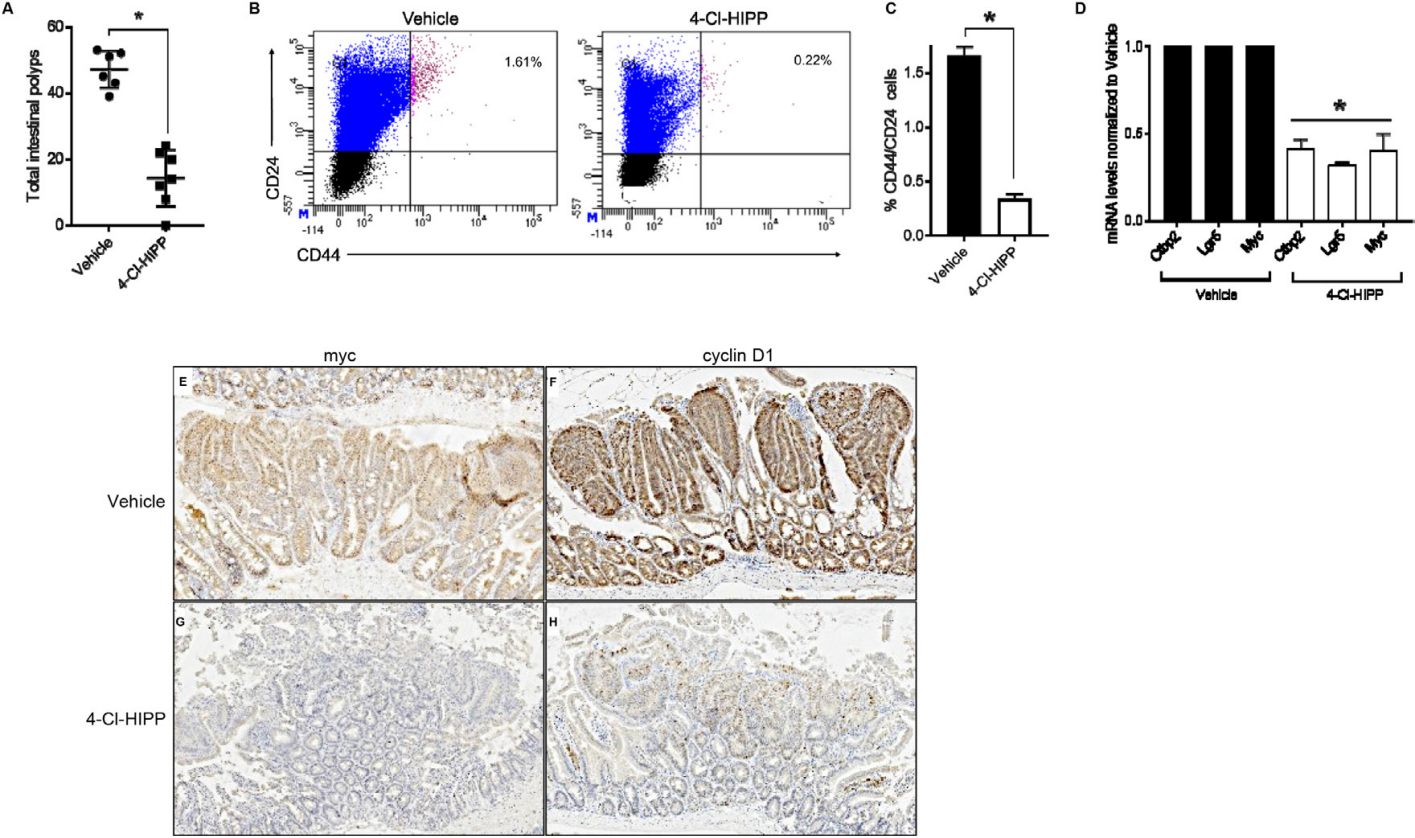
Pharmacological inhibition of Ctbp2 decreases polyposis and TIC populations in *Apc* ^*min/+*^ intestinal epithelia **(A)** Quantification of total intestinal polyps from Vehicle (n = 6) or 4-Cl-HIPP (n = 7) treated *Apc* ^*min/+*^ mice after 8 weeks of 4-Cl-HIPP treatment, p <0.02, error bars indicate 1 SD. **(B)** Scatter plot of representative flow cytometric analysis of intestinal epithelial cells with top right quadrants representing percentage of CD44+CD24+ cells in age matched mice treated with vehicle or 4-Cl-HIPP for 8 weeks. **(C)** Quantification of CD44+CD24+ population of cells in (B) (n= 4 biologic replicates); ^*^ p<0.02, error bars represent standard deviation from the mean. **(D)** mRNA levels of TIC-associated genes expressed in intestinal epithelial cells from vehicle or 4-Cl-HIPP treated mice; bars represent mean values from amplification of mRNA from intestinal epithelial cells obtained from 3 mice per group; ^*^p< 0.05, error bars represent standard deviation from the mean. **(E-H)** IHC staining of *Apc* ^*min/+*^ intestinal polyps from mice treated with vehicle or 4-Cl-HIPP, for c-Myc (E, G) or cyclin D1 (F, H).

As we have identified Ctbp2 as a key dependency for both *Apc* ^*min*^ intestinal polyposis and TIC populations, we investigated whether CtBP chemical inhibition, which can also suppress polyposis, likewise reduced TIC populations. The 1^st^ generation CtBP inhibitor, HIPP, targets the dehydrogenase domain of CtBP, diminishes CtBP function [2], and suppresses polyp formation by 50% in *Apc* ^*min*^ mice [2]. Given the high dose of HIPP required *in vivo*, we tested the most potent available HIPP derivative, 4-Cl-HIPP [18], for suppression of polyposis and effect on TIC populations in *Apc* ^*min*^ mice (Supplementary Figure 2). Previous studies performed with 4-Cl-HIPP suggest that it is a CtBP2 specific inhibitor due to its on-target ability to abrogate CtBP2-mediated transcriptional repression [18]. Using dosing with minimal toxicity to major organs (Supplementary Figure 2A), we saw that 4-Cl-HIPP was as effective as HIPP at suppressing polyposis at 8 weeks, with a 60% reduction noted, and at a dose 60% lower than HIPP. (Figure 4A, Supplementary Figure 2B-2C). Similar to the effect of *Ctbp2* haploinsufficiency, we observed a significant 5-fold decline in the percentage of dual positive CD44+CD24+ cells in intestinal epithelial cells of *Apc* ^*min*^ mice receiving 4-Cl-HIPP as compared to vehicle (Figure 4B-4C).

### Pharmacological inhibition of Ctbp blocks intestinal Wnt signalling

Colorectal cancers often exhibit aberrant activation of the Wnt/ βcatenin pathway leading to activation of downstream oncogenic targets, such as cyclin D1, c-Myc and the intestinal stem cell regulatory gene, Lgr5 [21, 25]. Since Ctbp2 plays a key role in Wnt/βcatenin mediated signaling by coactivating TCF4 [37], we sought to assess if pharmacological inhibition of Ctbp2 using 4-Cl-HIPP inhibits oncogenic Wnt signaling in *Apc* ^*min*^ intestinal epithelia, as we had previously found in *Ctbp2* haploinsufficient mice [2]. Indeed, the mRNA expression of c-Myc and Lgr5 was suppressed in 4-Cl-HIPP vs. vehicle-treated *Apc*^*min*^ intestinal cells (Figure 4D). 4-Cl-HIPP was also able to suppress Ctbp2 mRNA levels, possibly reflecting transcriptional autoregulation of the Ctbp2 gene by Ctbp2. The qPCR data was further supported by IHC of 4-Cl-HIPP vs. vehicle-treated *Apc* ^*min*^ small intestine, which revealed decreased c-Myc and cyclin D1 staining in polyps (Figure 4E-4H; stain intensity 3+ (vehicle) and 1+ (4-Cl-HIPP) for c-Myc and cyclin D1). Pharmacological inhibition and genetic ablation of Ctbp2 are therefore both associated with reduction in polyposis, diminished intestinal TIC populations, and reduction in Wnt pathway oncogenic signaling in *Apc* ^*min*^ intestine. Moreover, the concordance of genetic and pharmacologic data support 4-Cl-HIPP’s activity as reflecting on-target antagonism of Ctbp.

### CtBP2 ablation or pharmacologic inhibition attenuates TIC function and Wnt signalling in human colon cancer cells

**Figure 5:**
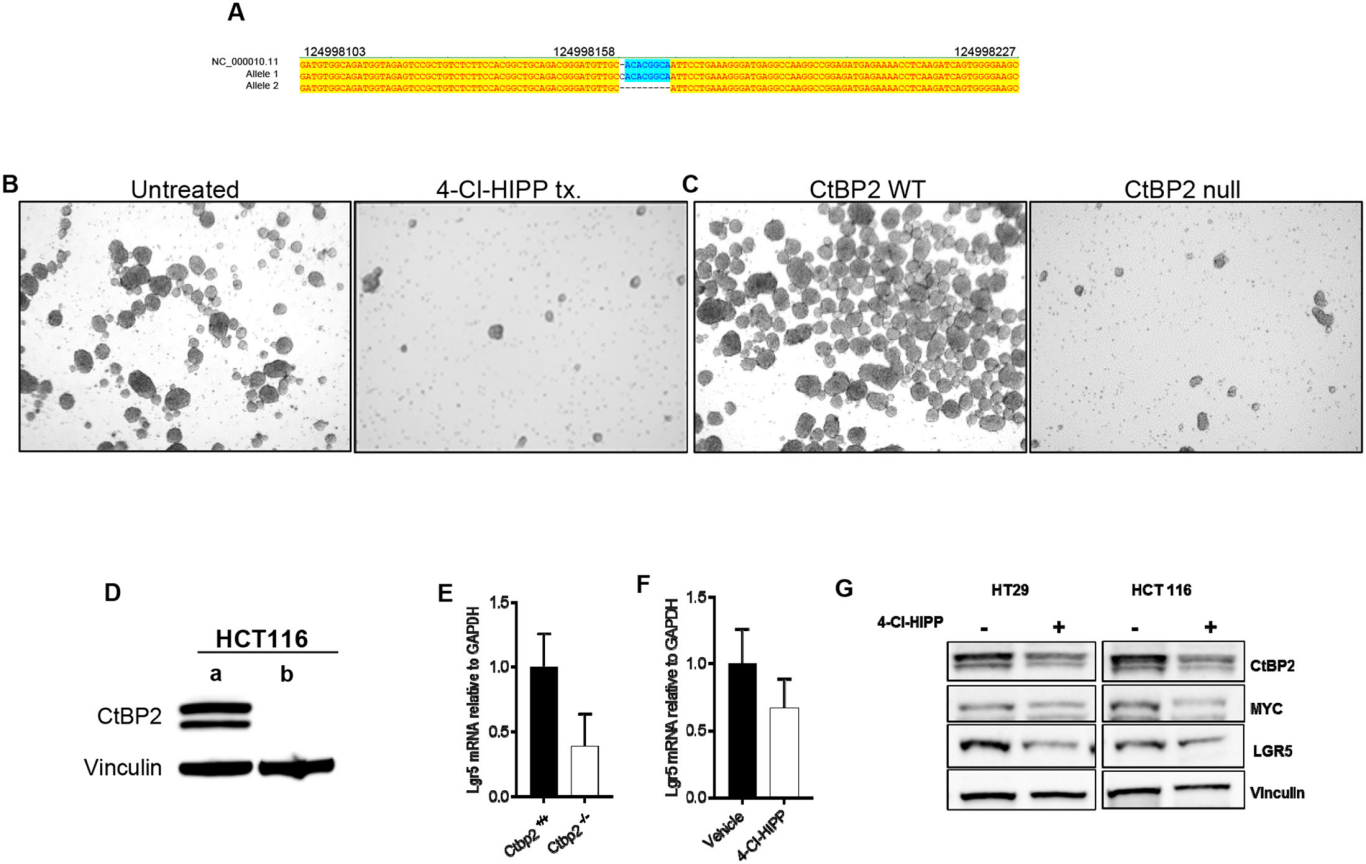
CtBP2 null human colon cancer cells have decreased TIC expression **(A)** ClustalW alignment of sequences of the HCT116 CtBP2 CRISPR knockout clone. Subcloned colonies from CRISPR targeting were sequenced using universal primer M13 F43, and representative clones aligned with wildtype *CtBP2* sequence. One allele has an insertion of one base pair while the other allele has a deletion of eight base pairs **(B)** HCT116 primary tumorspheres treated with vehicle or 4-Cl-HIPP after 5 days of incubation. **(C)** HCT116 tumorspheres prepared from parental (left) or CtBP2 null clone (right) after 5 days in culture. **(D)** Immunoblot analysis of CTBP2 expression in HCT116 (a) parental cells, and (b) CTBP2 null sub-clone from (A). Vinculin was a loading control. **(E, F)** Relative *Lgr5* mRNA levels in (E) HCT116 parental vs. *CtBP2*-null tumorspheres or (F) HCT116 tumorspheres treated with vehicle or 4-Cl-HIPP. **(G)** Immunoblot of vehicle vs. 4-Cl-HIPP treated HT29 and HCT116 tumorsphere lysates using indicated antibodies.

We have illustrated efficient 4-Cl-HIPP inhibition of intestinal TIC populations in the *Apc* ^*min*^ polyposis (pre-cancer) model. To determine if 4-Cl-HIPP could similarly inhibit human TIC populations in cancer cells, and mirror effects of CtBP2 deficiency, we utilized human colorectal cancer cell lines that form tumor spheroids (tumorspheres) that are enriched for TIC’s [19] to assay the effect of CtBP inhibition or CtBP2 genetic depletion. We generated HCT116 colon cancer cells genetically deficient for *CtBP2* using CRISPR knockout techniques (Figure 5A, 5D), and compared the effect of 4-Cl-HIPP treatment vs. *CtBP2* knockout on tumorsphere growth. Notably, 4-Cl-HIPP efficiently disrupted primary sphere formation of HCT116 cells (Figure 5B). Additionally, HCT116 cells with CRISPR-mediated deletion of both *CtBP2* alleles were also unable to form primary tumorspheres (Figure 5C), suggesting tumorsphere inhibition, and by inference, TIC inhibition, is an on-target activity of 4-Cl-HIPP.

Translating our TIC marker and Wnt pathway results from *Apc* ^*min*^ mouse intestinal epithelia to human colon cancer cells, and consistent with CtBP’s regulation of TIC-enriched tumorspheres, LGR5 mRNA and/or protein expression was also effectively disrupted by *CtBP2* knockout in HCT116 tumorspheres (Figure 5E), or by 4-Cl-HIPP treatment in HCT116 or HT29 tumorspheres (Figure 5F-5G). Along with LGR5 regulation, we have demonstrated that *Ctbp2* deficiency is associated with downregulation of Wnt pathway oncogenic effectors [2] and 4-Cl-HIPP likewise inhibited downstream Wnt pathway targets c-Myc and cyclin D1 in *Apc*^*min*^ polyps (Figure 4E-4H). We therefore examined the effect of 4-Cl-HIPP on c-Myc expression in HCT116 tumorspheres, as c-Myc is a downstream oncogenic target of the Wnt/ TCF4 pathway [38], and observed a robust decrease in c-Myc protein levels after 4-Cl-HIPP treatment (Figure 5G). Thus, 4-Cl-HIPP can phenocopy CtBP2 knockout and inhibit the Wnt target and TIC marker LGR5, and also attenuate expression of the key oncogenic Wnt pathway target c-Myc in human colon cancer cells.

## DISCUSSION

Years of study in cell culture and in human tumors have implicated CtBP1 and 2 as oncogenic, by virtue of observed activities in TIC regulation, EMT induction, chemoresistance and common overexpression in solid tumors linked to poor outcome [39]. Our recent observations that CtBP2 is a key dependency in *Apc*-mutated neoplasia, was the first *in vivo* evidence of CtBP2’s neoplastic potential [2]. In this work, we have determined the biologic basis of CtBP2’s neoplastic activities in *Apc-*mutated neoplasia as linked to its regulation of TIC populations and transcriptional regulation of downstream Wnt targets, such as LGR5, c-Myc and cyclin D1. Moreover, relative deficiency of Ctbp2, or functional inhibition, in the early progenitor niche in crypts, may suppress *Apc*-deficiency associated neoplasia, as Ctbp2 is a key component of the pathway between *Apc* loss and neoplastic transformation, via its regulation of c-Myc and cyclin D1.

The surprising findings relative to Ctbp2’s unique cytoplasmic localization when genetically haploinsufficient, could explain the drastically lower TIC and polyp count in *Ctbp2*^*+/-*^ *Apc*^*min/+*^ mice, as Ctbp2 localized in the cytoplasm would be partially or fully inactive to perform transcriptional coregulation of Wnt target genes [40]. As a result, crucial Wnt targets that play a driver role in cell proliferation and TIC function, such as c-Myc and cyclin D, would be expressed at lower levels, as already observed in *Ctbp2* haploinsufficient *Apc* ^*min*^ polyps [2], and thus render stem cell precursors to TIC’s less likely to transform. Cytoplasmic accumulation of Ctbp2 in the stem cell zone of normal (non-adenomatous) crypts, along with partial loss of CD133 in the setting of Ctbp2 haploinsufficiency, is evidently compatible with normal intestinal development, but could have implications in slower recovery of intestinal crypt regeneration and higher sensitivity of the gut to stress or injury, as the stem cell reserve may be limited. Still unclear, is exactly why Ctbp2 is found in the cytoplasm when it is haploinsufficient, which requires further study, but may be related to stoichiometry of its binding to APC protein (which like Ctbp2, also exhibits a dot like pattern in the cytoplasm [41]). Thus, Ctbp2 is potentially a key driver of transformation of normal crypt CD133+ stem cells to adenomatous polyps, and thus, a driver of the intestinal TIC phenotype in *Apc* mutated intestinal neoplasia.

Our evaluation of the 2^nd^ generation CtBP inhibitor 4-Cl-HIPP demonstrates that targeting CtBP is safe and efficacious in the *Apc* ^*min*^ model, and moreover, its effect on TIC populations *in vivo* and *in vitro* phenocopies *Ctbp2* knockout, consistent with on-target activity. Further evaluation of 4-Cl-HIPP and related inhibitors, alone or in combination with standard therapies, is warranted in colorectal cancer. Our data also suggests that anti-CtBP therapy, in general, may serve as a novel anti-TIC therapy in settings where TIC’s represent a mechanism for cancer relapse and/or chemoresistance [42].

## MATERIALS AND METHODS

### Cell culture and sphere assay

HCT116 cells and HT29 cells (ATCC) were maintained in RPMI 1640 medium in tissue culture treated plates and passaged using trypsin-EDTA upon 70% confluency. For tumorsphere assays, stem cell media (SCM) was prepared as follows; DMEM/ F12 media supplemented with 1% penicillin/ streptomycin, 20ng/ml epidermal growth factor, 10ng/ml fibroblast growth factor and B27 were used. Tumorsphere culture was maintained using 200 cells/ well of an ultra-low attachment plate in SCM and measured for protein and mRNA at the end of 5 days. For primary tumorspheres, cells were seeded in SCM on day one and passaged on day 5 for secondary tumorspheres. On day 10, tumorspheres were passaged for tertiary spheres and harvested on day 15.

### Western blotting

Cells were washed in cold PBS and lysed in RIPA buffer (150mM NaCl, 50mM Tris HCL, pH 8.0, 1.7% NP-40, 0.17% sodium dodecyl sulfate (SDS), 0.5% Na-deoxycholate monohydrate, 5mM EDTA) containing 1 tablet of complete Mini Protease Inhibitor Cocktail /10ml (Roche). Lysates were cleared of insoluble material by centrifugation at 15,000 RPM. Proteins (30 μg) were loaded onto a Bis-Tris 4-12% gel containing NuPAGE MOPS buffer. Antibodies- Ctbp2 (Cat no. 612044, BD transduction); c-Myc (N-262; Cat no. sc-764, Santa Cruz).

### Real-time polymerase chain reaction (qPCR) analysis

Total RNA was isolated using RNeasy (Qiagen) and manufacturer’s protocol was followed. 1μg total RNA was reverse transcribed using sensiFAST cDNA (Bioline). The cDNA was amplified in triplicate using SYBR green and the specific primers were used to detect amplicons. The primers used for qPCR were as follows human CtBP2 F 5'- ATCCACGAGAAGGTTCTAAACGA -3', R- 5'CCGCACGATCACTCTCAGG -3'; human LGR5 F- 5'- GGAGGAGGGAGAACCCACTT -3', R- 5'-TCCCATGGATCACAGCCTCT -3'; mouse Ctbp2 F- 5'- GGCAAGGTGCATTCCTTGTG -3', R- 5'- TCGTATCCTGCCCTCCTTGA -3'; Mouse Lgr5 F- 5'- ACCAGCTTACCCCATGACTG -3', R- 5'- CTCCTGCTCTAAGGCACCAC -3'.

### Mice

All animal studies performed for this manuscript were approved by VCU Institutional Animal Care and Use Committee. The C57BL/6J male mice heterozygous for the *Apc Min* allele (*Apc* ^*min/+*^) and C57BL/6J male mice heterozygous for the *Ctbp2* allele (*Ctbp2* ^*+/-*^) were purchased from Jackson Laboratory (Bar Harbor, ME). Each genotype was established by mating the founder male with wild-type C57BL/6J female mice with further backcross of the mutant genotypes to C57BL/6J at least 6 generations before initiating experiments. Allele-specific PCR assays were used to identify the *Apc Min* and the *Ctbp2* mutations (end point PCR: *Apc* ^*min/+*^, F- 5′-GGGAAGTTTAGACAGTTCTCGT-3′and R-5′-TGTTGGATGGTAAGCACTGAG-3′, Mutant- 5′- AGACAGAAGTTAGGAGAGAGAGC-3′and WT- 5′-AGACAGAAGTTTGGAGAGAGAGC-3′. *Ctbp2* ^*+/−*^ Internal Control, F- 5′-CAAATGTTGCTTGTCTGGTG-3′and R- 5′-GTCAGTCGAGTGCACAGTTT-3′, Mutant F-5′-CCAGTGGGGATCGACGGTATC-3′, Mutant R- 5′-CACTCCAACGCAGCACCATC-3′). All mice were fed regular water and chow and optimum day and night cycles were maintained.

### Mouse small intestinal mucosal cell isolation

Mouse small intestines were isolated and flushed with HBSS with 2% glucose solution 2 times in closed conformation. Intestinal slices were cut longitudinally along the length and cleaned for debris and fecal matter. The intestines were cut and chopped into fine pieces of about 2mm in size and stored in HBSS+2% glucose on ice. Tissue was resuspended in HBSS-glucose-dispase-collagenase solution and placed on a shaker for 25 min at 25°C. The digested tissue was further disaggregated by hand pipetting for 3 min. This was followed by 3 slow centrifugations at 300rpm with resuspension of pellets in HBSS+2% glucose to further purify the intestinal mucosal cell population.

### Flow cytometry

Isolated mouse small intestinal mucosal cells were incubated with conjugated antibody for 30 minutes at 4°C and washed with PBS buffer prior to analysis. The following antibodies and dilutions were used: CD133/1 (AC133)- APC (1:33 dilution) (Miltenyi Biotec, Auburn, CA, USA); CXCR4-PE conjugated clone 2B11 (Dilution 1:50) (BD Pharmingen, San Jose, CA, USA); CD24- PE conjugated clone M1/69 (BD Pharmingen). Stained cells were run in the Fortessa flow cytometer (BD Biosciences, San Jose, CA, USA) and data were analyzed using FCS Express 4 Flow Research Edition software (De-Novo Software, Glendale, CA, USA) and FACS diva (BD biosciences).

### Immunohistochemistry and immunofluorescence

Mouse intestinal swiss rolls were formalin fixed and paraffin embedded on slides and were subjected to deparaffinization and hydration steps followed by quenching and peroxidase reaction steps [43]. Antigen retrieval was performed using a pressure cooker, followed by blocking for an hour 5% goat serum and incubation with primary antibodies at a dilution of 1:100 in blocking buffer with CtBP2 (Cat no. 612044, BD Biosciences); CD133 (Cat no. 18470-1-AP, Proteintech, Rosemont, IL, USA); c-Myc (N-262, Cat no. sc-764, Santa Cruz Biotechnology, SantaCruz, CA, USA); cyclin D1 (DCS-6, Cat no. 20044, Santa Cruz Biotechnology) overnight. After 3 washes in PBS, secondary antibodies (HRP conjugated anti mouse or anti rabbit IgG, Cat no. K4000, Dako Envision Systems, Santa Clara, CA, USA) were incubated for 1 hour followed by 3 washes in PBS and slide development using DAB chromogen substrate from Dako Envision Systems (Cat no. K3467). Nuclear staining was performed by incubation in hematoxylin for 2 mins and was followed by dehydration and cover-slipping steps. For IF, steps were similar to IHC, except the secondary antibodies used for detection of CD133 and CtBP2 were anti rabbit (Alexa fluor 594; Cat# A11012; Invitrogen) and anti mouse (Cruz fluor 488; Cat# sc-362257; Santa Cruz Biotechnology), respectively, followed by similar wash steps as IHC. After dehydration steps, slides were mounted with mounting media containing DAPI (Cat no. S36938, Invitrogen, Eugene, OR, USA) and air dried before analysis.

### CRISPR/Cas9-mediated deletion of *CtBP2* in HCT116 cells

Annealed DNA oligonucleotides DP225-5'- TGC AGA CGG GAT GTT GCA CAG TTT T -3'; DP226- 5'- TGT GCA ACA TCC CGT CTG CAC GGT G -3') that coded for the target specific crRNa were ligated with linear GeneArt CRISPR Nuclease Vector with Orange Fluorescent Protein (OFP) (Cat No: A21174; Thermo Fisher Scientific, Waltham, MA, USA). Electrocompetent *E.coli (DH10B)* were transformed and grown overnight on LB-Agar plates supplemented with 100 μg/ml Ampicillin. Individual clones were purified by streaking and their plasmid DNA was verified by sequencing. HCT116 cells at 70% confluency, grown in RPMI 1640 supplemented with 10% FBS, in a 100mm dish were transfected with 6μg DNA using Lipofectamine 2000. The cells were incubated at 34°C (5% CO_2_) for 72 hours and OFP positive cells were single cell fluorescence activated cell sorted in a 96 well plate using Aria-BD, (San Jose, CA, USA) FACSAria™ II High-Speed Cell Sorter at λ_exc_=488nm. The cells were allowed to grow to confluency before splitting them in triplicate plates to screen for mutant clones.

Clones were washed in cold PBS and lysed in 96 well plates using 50 μL RIPA buffer supplemented with Protease inhibitor cocktail (Roche). Samples were clarified by centrifugation, prepared for SDS PAGE, and resolved on 15 well Bis-Tris Gradient polyacrylamide gels (4-12%) (NuPAGE, Thermo Fisher Scientific) using MOPS as a running buffer followed by a wet transfer to a 0.45μm PVDF membrane (Immobilon-FL, CAT. NO: IPFL00010, Millipore). The membranes were incubated overnight with Anti-CtBP2 Mouse mAb (Catalog No.612044 BD Biosciences) and anti-Vinculin (E1E9V) Rabbit mAb (Cell Signalling, Beverly, MA, USA) followed by 1h incubation with Alexafluor (680) labelled secondary antibodies (Thermo Fisher Scientific). The blots were scanned at 685nm (Odyssey CLx scanner, Odyssey, Lincoln, NE, USA).

After screening, mutant clones were grown to confluency in 6 well plates and genomic DNA was extracted using ISOLATE II Genomic DNA Kit (Cat No. BIO-52067, Bioline, Taunton, MA, USA) as per the manufacturer’s instructions. The mutated region of the alleles was PCR amplified using oligonucleotides DP261 and DP262 (DP261-5'- CTG CCA GCT CCA TGA GGG AG -3'; DP262- 5'- CAG GCT GGG GTT GCC TTT CC -3') with Taq Polymerase (Cat No: BIO-25043, MyTaq Red Mix, Bioline), purified using Qiagen PCR purification kit and ligated with pGEM®-T Vector (Cat No: A3600, Promega, Madison, WI, USA). Electrocompetent *E.coli (DH10B)* were transformed with the ligation mix and blue-white screening was performed. White colonies were grown overnight; plasmid DNA was extracted and sequenced to confirm mutations.

## SUPPLEMENTARY MATERIALS FIGURES



## Abbreviations

CtBP2: C terminal binding protein 2
Apc^min^: Adenomatous Polyposis Coli Multiple Intestinal Neoplasia
TIC: Tumor initiating cells
FAP: Familial Adenomatous Polyposis
CRISPR: Clustered Regularly Interspaced Short Palindromic Repeats
HIPP: 2-hydroxyimino-3-phenyl-propionic acid
MTOB: α-keto-γ-(methylthio) butyric acid
