# Supplementary material for: An intestinal stem cell niche in Apc mutated neoplasia targetable by CtBP inhibition
Source: Oncotarget. 2018 Aug 21;9(65):32408–18. doi: 10.18632/oncotarget.25784 (PMC6126694; doi:10.18632/oncotarget.25784)
Supplement: Supplementary file 1 [file oncotarget-09-32408-s001.pdf]

An intestinal stem cell niche in *Apc* mutated neoplasia targetable by CtBP inhibition

SUPPLEMENTARY MATERIALS

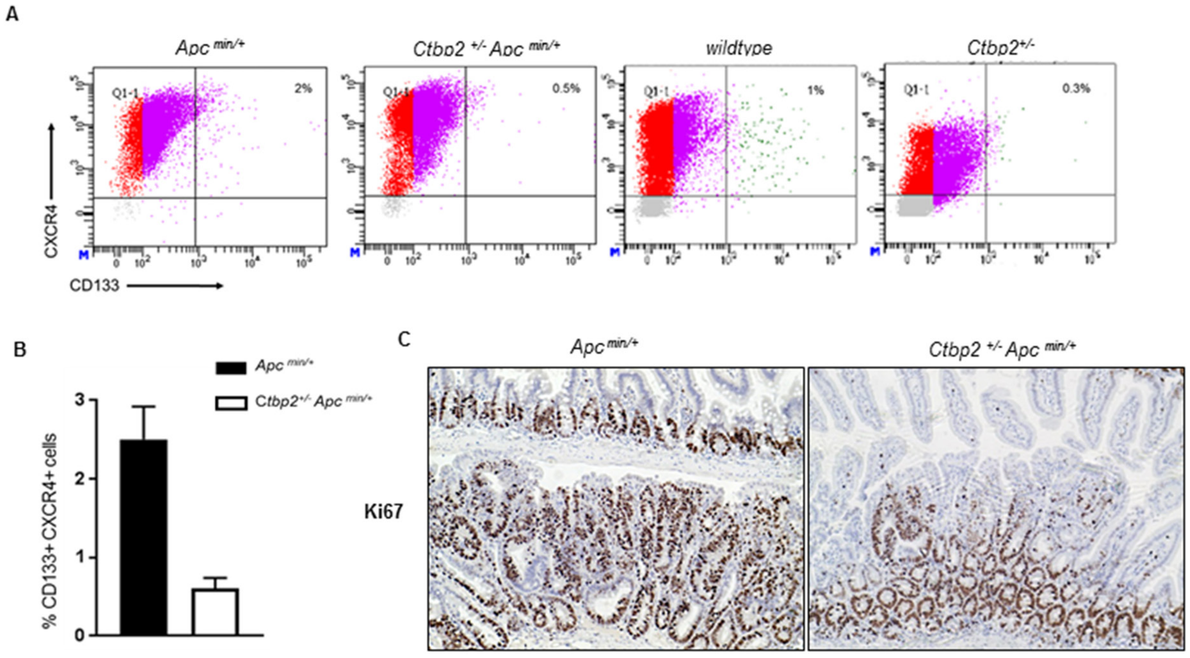

Supplementary Figure 1: Ctbp2 haploinsufficiency decreases TIC populations in *Apc*$^{min/+}$ intestinal epithelia. **(A)** Scatter plot of representative flow cytometric analysis of intestinal epithelial cells with top right quadrants representing percentage of CD133+/CXCR4+ cells in age-matched mice (4 months) of indicated genotypes. **(B)** Quantification of CD133+CXCR4+ cells from intestinal epithelia of indicated genotypes; n=3 biologic replicates. **(C)** Ki67 showing proliferating cells, as detected by IHC staining, in indicated genotypes.

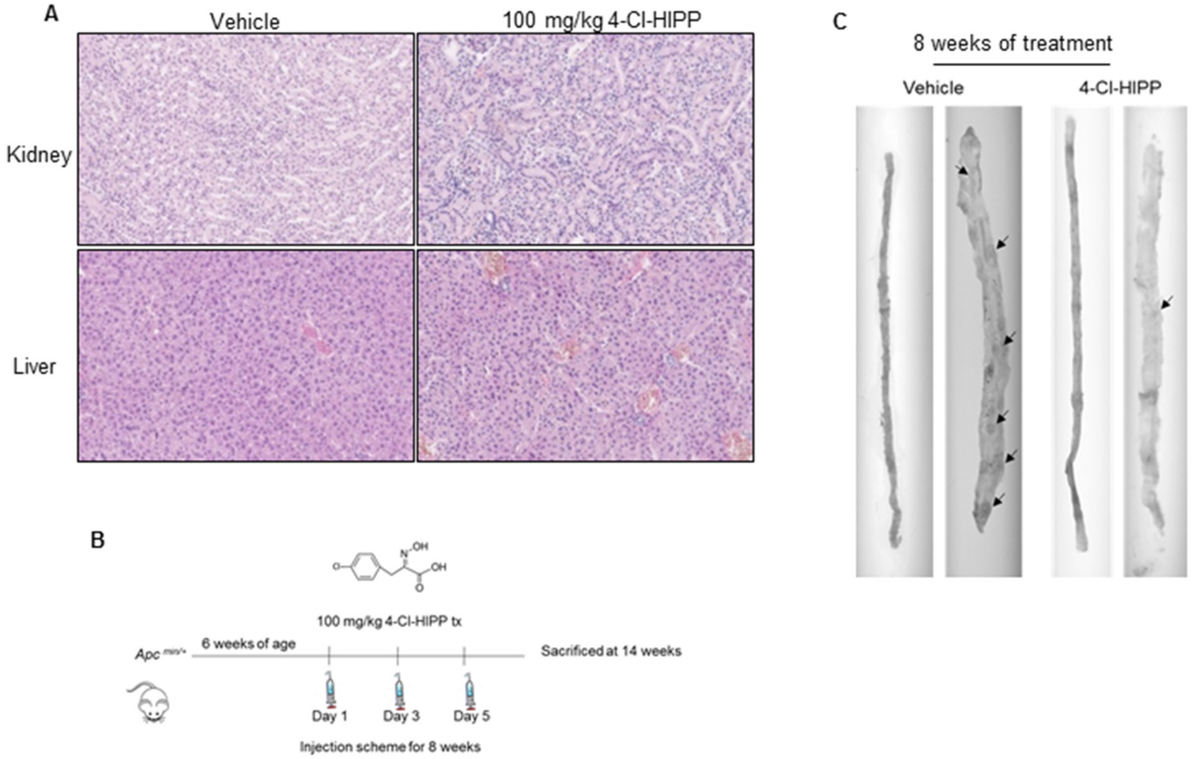

Supplementary Figure 2: (A) Hematoxilin and eosin (H&E) sections of kidney and liver from wildtype mice (6 weeks of age) treated with 100mg/kg 4-Cl-HIPP administered intraperitoneally (IP) 3 times per week for 6 weeks. **(B)** Treatment scheme for *Apc* $^{min/+}$ mice at 6 weeks of age. 4-Cl-HIPP was injected at 100 mg/kg IP 3x per week for 8 weeks, and mice were then euthanized 48h after the last injection. **(C)** Closed (left) and open (right) conformations of *Apc* $^{min/+}$ mouse small intestine harvested at age 14 weeks after 8 weeks of treatment with vehicle or 4-Cl-HIPP.
